# Nonalcoholic Wernicke Encephalopathy Following Prolonged Intermittent Fasting: A Case Report

**DOI:** 10.7759/cureus.102314

**Published:** 2026-01-26

**Authors:** Anas E Ahmed, Arwa O Alharthi, Abdullah H Al-Hm‎oad, Weaam A Felemban, Adhwaa S Abdulhakeem

**Affiliations:** 1 Community Medicine, Jazan University, Jazan, SAU; 2 College of Medicine, University of Bisha, Bisha, SAU; 3 College of Medicine, King Faisal University, Al Hofuf, SAU; 4 Emergency Medicine, Al-Shimeisy Medical Complex, Makkah, SAU; 5 Emergency Medicine, King Abdulaziz Hospital, Makkah, SAU

**Keywords:** encephalopathy, intermittent fasting, nonalcoholic, nutritional deficiency, thiamine deficiency, weight loss, wernicke encephalopathy

## Abstract

Wernicke encephalopathy (WE) is an acute and potentially reversible neurological disorder caused by thiamine deficiency and is most commonly associated with chronic alcohol use. However, it can also occur in nonalcoholic individuals with nutritional deprivation, where diagnosis is often delayed because of low clinical suspicion. We report a case of WE occurring in the setting of prolonged intermittent fasting for weight loss. The patient presented with subacute neurological symptoms, including gait disturbance, visual complaints, and cognitive impairment. Neurological examination revealed findings consistent with encephalopathy, including ocular motor abnormalities and ataxia. Routine laboratory investigations were largely unremarkable, aside from evidence of nutritional deficiency. Initial neuroimaging did not reveal acute abnormalities, while subsequent magnetic resonance imaging (MRI) demonstrated changes compatible with WE. A detailed dietary history identified prolonged fasting with inadequate nutritional intake as the likely contributing factor. The patient was treated with parenteral thiamine and supportive nutritional management, resulting in clinical improvement. This case emphasizes the importance of recognizing nonalcoholic causes of WE in the context of restrictive dietary practices. Early recognition and prompt thiamine replacement are essential to prevent long-term neurological complications, underscoring the need for increased clinical awareness as fasting-based dietary approaches become more common.

## Introduction

Wernicke encephalopathy (WE) is an acute, potentially life-threatening but reversible neurological disorder resulting from thiamine (vitamin B1) deficiency [1,2]. Thiamine is an essential cofactor in cerebral glucose metabolism, and its deficiency leads to impaired energy production, oxidative stress, and selective neuronal vulnerability in metabolically active brain regions [2,3]. Classically, WE is associated with chronic alcohol use; however, growing evidence indicates that a substantial proportion of cases occur in nonalcoholic individuals [1-3]. In such patients, WE is frequently underdiagnosed due to lower clinical suspicion and the absence of traditional risk factors, increasing the risk of delayed treatment and permanent neurological sequelae.

Nonalcoholic WE has been reported in association with malnutrition, prolonged fasting, gastrointestinal disease, bariatric surgery, hyperemesis, and eating disorders [2,3]. In recent years, intermittent fasting has gained widespread popularity as a weight loss strategy, often perceived as safe and health-promoting [1-4]. While moderate fasting regimens may be well tolerated, extreme or prolonged fasting without adequate nutritional supplementation can lead to critical micronutrient deficiencies, including thiamine depletion. Given the limited body stores of thiamine and its rapid depletion during periods of inadequate intake, individuals engaging in aggressive fasting practices may be particularly vulnerable [2,5]. This case highlights prolonged intermittent fasting as an underrecognized precipitant of nonalcoholic WE and underscores the importance of early recognition, dietary history taking, and prompt thiamine replacement in preventing irreversible neurological injury.

Furthermore, the rising global popularity of intensive dietary modifications for weight management, particularly various forms of intermittent fasting, has introduced a new and under-recognized risk factor for acute nutritional deficiencies [2-6]. While these practices are often perceived as benign, the combination of prolonged caloric restriction and potential lack of dietary diversity can rapidly exhaust the body’s limited thiamine reserves, which are not synthesized endogenously and last only two to three weeks. This creates a vulnerable window for the development of WE, even in otherwise healthy, nonalcoholic individuals [2-6].

## Case presentation

A 34-year-old woman with no significant past medical history presented to the emergency department with a 10-day history of progressive neurological symptoms. The patient reported intermittent dizziness, unsteady gait, blurred vision, and increasing difficulty with concentration and short-term memory. Over the preceding three days, her family noticed confusion, apathy, and slowed responses. There was no history of alcohol consumption, substance abuse, chronic liver disease, gastrointestinal surgery, hyperemesis, or use of diuretics. She denied fever, headache, seizures, focal weakness, sensory loss, bowel or bladder dysfunction, or recent infection.

Of note, the patient reported intentional weight loss over the preceding four months through prolonged intermittent fasting, consisting of fasting periods of 48-72 hours repeated weekly with minimal caloric intake on non-fasting days. She reported an approximate weight loss of 18 kg during this period and acknowledged poor dietary diversity and minimal vitamin supplementation.

On initial examination, the patient was afebrile, with a blood pressure of 118/72 mmHg, a heart rate of 84 beats per minute, a respiratory rate of 16 breaths per minute, and an oxygen saturation of 99% on room air. She appeared thin but not cachectic. Neurological examination revealed an alert but inattentive patient, oriented to person and place but inconsistently to time. Speech was slow but coherent. Cranial nerve examination demonstrated bilateral horizontal nystagmus, more pronounced on lateral gaze, with mild bilateral ophthalmoplegia affecting abduction. Pupils were equal and reactive, and fundoscopic examination was unremarkable. Motor examination showed normal bulk and tone with full strength in all extremities. Sensory examination was intact to light touch and proprioception. Coordination testing revealed dysmetria on finger-to-nose testing and marked truncal ataxia. Gait was wide-based and unstable, requiring assistance. Deep tendon reflexes were normal and symmetric, and plantar responses were flexor. The remainder of the physical examination was unremarkable.

Initial laboratory investigations demonstrated mild normocytic anemia (hemoglobin 11.2 g/dL), with normal white blood cell count and platelet count. Serum electrolytes, renal function, liver enzymes, thyroid-stimulating hormone, and blood glucose levels were within normal limits. Inflammatory markers were not elevated. Serum vitamin B12 and folate levels were within reference ranges. Serum magnesium was mildly reduced (1.5 mg/dL; reference 1.7-2.2 mg/dL). Serum thiamine (vitamin B1) level was significantly decreased at 38 nmol/L (reference 70-180 nmol/L). Urine toxicology screening was performed via standardized immunoassay and was negative for amphetamines, barbiturates, benzodiazepines, cocaine metabolites, and opioids. Lumbar puncture was not performed due to low suspicion for infectious or inflammatory etiologies.

**Figure 1:**
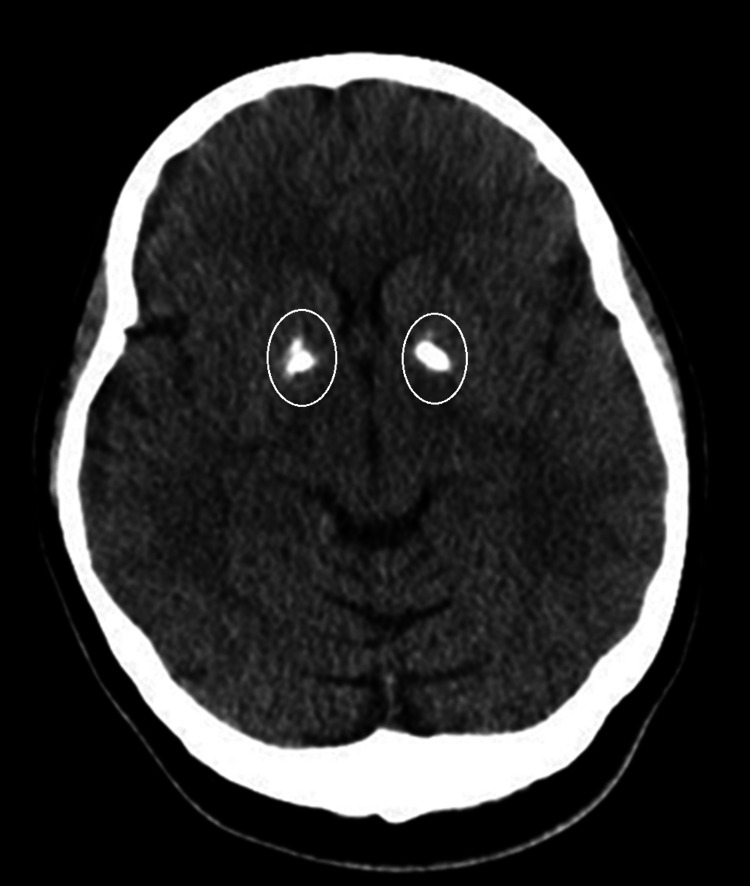
Axial non-contrast CT of the brain An axial CT image of the brain shows normal parenchymal structures. There is basal ganglia calcification (encircled), a common benign finding, with no associated mass effect or acute hemorrhage.

**Figure 2:**
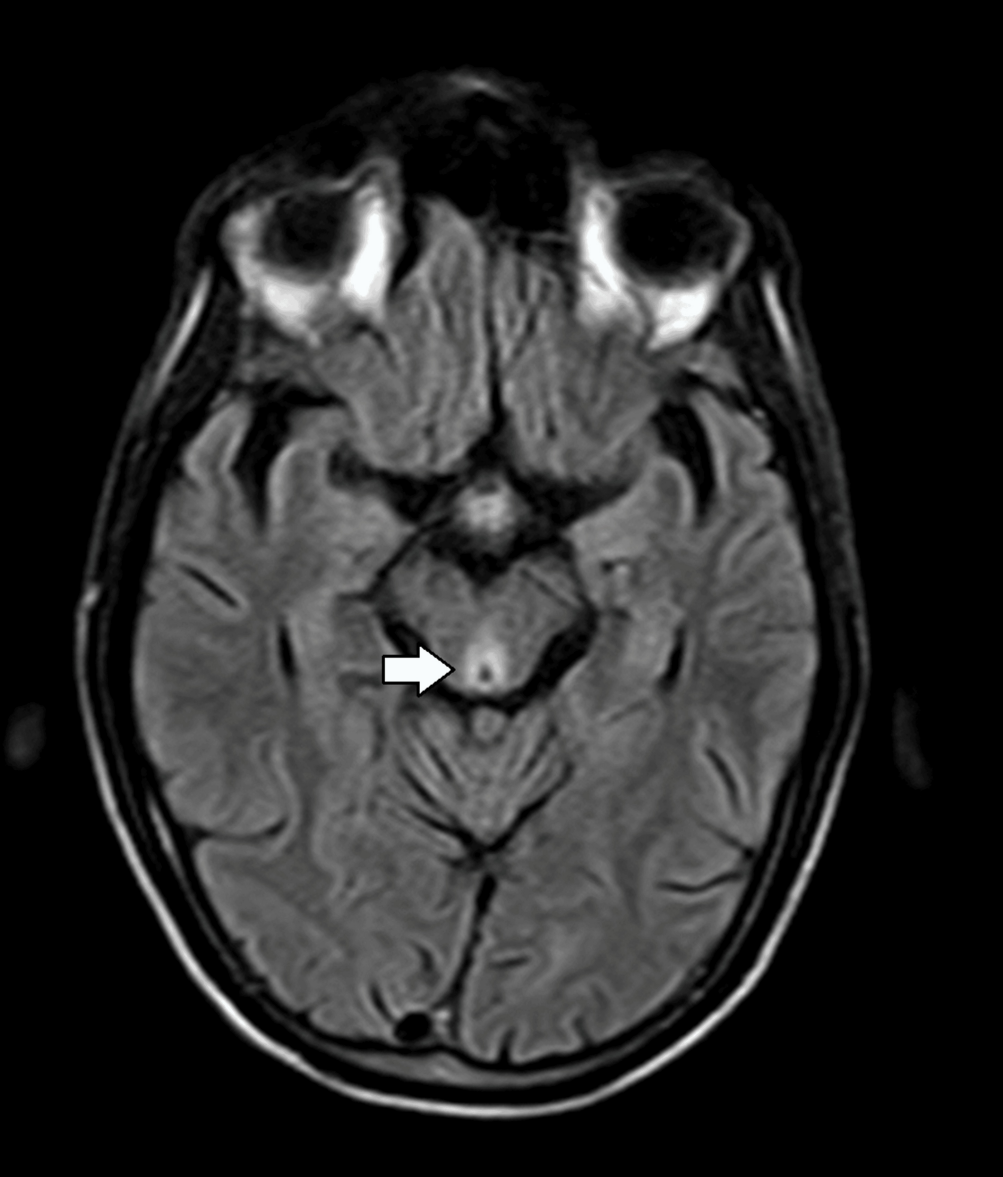
Axial FLAIR magnetic resonance image of the brain The axial FLAIR image shows a hyperintense signal in the white matter surrounding the third ventricle (arrow), consistent with involvement of periventricular regions commonly affected in WE. FLAIR: fluid-attenuated inversion recovery; WE: Wernicke encephalopathy

**Figure 3:**
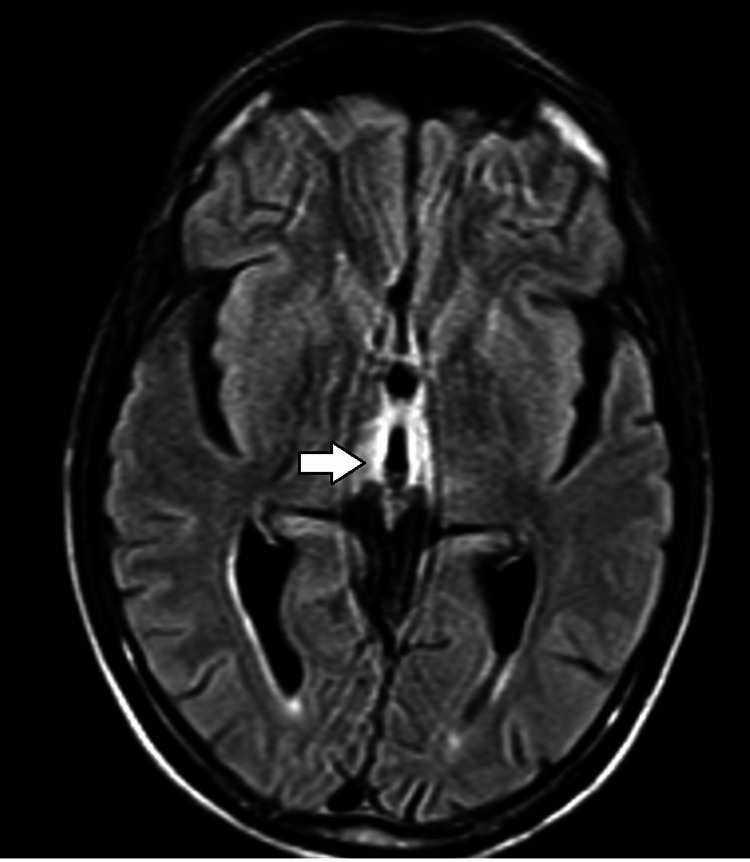
Axial FLAIR magnetic resonance image of the brain The axial FLAIR image demonstrates symmetric hyperintensity in the periaqueductal gray matter (arrow), a region surrounding the cerebral aqueduct. This finding is characteristic of WE. FLAIR: fluid-attenuated inversion recovery; WE: Wernicke encephalopathy

A non-contrast CT scan of the brain performed on admission was unremarkable, with no evidence of acute ischemia, hemorrhage, mass effect, or hydrocephalus. Incidental bilateral basal ganglia calcifications were noted and interpreted as benign and unrelated to the presenting symptoms (Figure 1). Given the strong clinical suspicion for metabolic or nutritional encephalopathy, an MRI of the brain was obtained. MRI demonstrated symmetrical hyperintense signal abnormalities on T2-weighted and fluid-attenuated inversion recovery (FLAIR) sequences involving the medial thalami (Figure 2), mammillary bodies, periaqueductal gray matter, and tectal plate (Figure 3). These regions also showed restricted diffusion on diffusion-weighted imaging without significant contrast enhancement, findings considered classic for WE.

The patient was promptly treated with high-dose parenteral thiamine (500 mg intravenously three times daily) initiated immediately after clinical suspicion, before glucose administration. Intravenous magnesium supplementation was also provided. After three days, thiamine was reduced to 250 mg intravenously once daily for an additional five days, followed by a transition to oral thiamine 100 mg three times daily. Nutritional rehabilitation was initiated with dietitian involvement, emphasizing gradual caloric repletion and balanced micronutrient intake. No corticosteroids or antimicrobial therapies were administered.

During hospitalization, the patient showed significant and objective clinical improvement following the initiation of high-dose intravenous thiamine and magnesium. Neurological recovery followed a distinct timeline: by hospital day two, her horizontal nystagmus had resolved, and ocular motility was full without diplopia. Her mental status showed marked progress; she became fully oriented to person, place, and time, and serial cognitive bedside assessments demonstrated normalized attention and short-term recall. By hospital day four, the truncal ataxia had improved considerably, allowing her to sit unassisted. Coordination testing revealed only mild residual dysmetria. Gait training with physical therapy began successfully, transitioning from a wide-based, two-person assist gait to a narrow-based gait with a single-person assist by day six. Supporting laboratory evidence included the normalization of serum magnesium to 2.0 mg/dL within 48 hours of supplementation. While serum thiamine levels were not rechecked during the acute admission to avoid interrupting therapy, a follow-up measurement six weeks post-discharge confirmed normalization at 145 nmol/L. This objective progression, from a state of global neurological dysfunction to rapid resolution of hallmark signs, correlated directly with treatment and provided clear, measurable substantiation of her significant clinical improvement.

At outpatient follow-up six weeks later, the patient reported complete resolution of visual symptoms and cognitive complaints. Neurological examination showed normal coordination and gait. Repeat serum thiamine levels had normalized. She was counseled extensively on safe weight loss practices and the risks associated with prolonged fasting.

## Discussion

WE is a neurologic emergency caused by thiamine deficiency and remains widely underrecognized, particularly in nonalcoholic populations [1-6]. Although classically associated with chronic alcohol use, up to half of reported cases occur in patients without alcoholism, often leading to diagnostic delay and worse neurological outcomes [2,3]. This case contributes to the growing body of evidence that extreme dietary practices, including prolonged intermittent fasting, can precipitate WE even in young, otherwise healthy individuals. As fasting-based weight loss strategies gain popularity, clinicians must broaden their awareness of nutritional encephalopathies beyond traditional risk groups.

Thiamine is an essential water-soluble vitamin that functions as a cofactor for enzymes involved in glucose metabolism, including pyruvate dehydrogenase, α-ketoglutarate dehydrogenase, and transketolase [1,2]. Deficiency leads to impaired cerebral energy utilization, lactate accumulation, oxidative stress, and neuronal injury, particularly in regions with high metabolic demand. The selective vulnerability of the medial thalami, mammillary bodies, periaqueductal gray matter, and tectal plate explains both the clinical manifestations and the characteristic MRI findings observed in WE [3-5]. Importantly, body thiamine stores are limited and may be depleted within two to three weeks of inadequate intake, making individuals engaging in prolonged fasting particularly susceptible.

The clinical diagnosis of WE remains challenging. The classic triad, such as encephalopathy, ocular motor dysfunction, and ataxia, is present in fewer than 20% of cases, and reliance on this triad alone contributes to underdiagnosis [2-6]. In nonalcoholic WE, presentations may be even more subtle, with cognitive changes or gait instability predominating. In the present case, the absence of alcohol use, normal initial CT imaging, and relatively nonspecific early symptoms could have diverted attention toward alternative diagnoses such as stroke, demyelinating disease, or autoimmune encephalitis [1,5]. However, careful dietary history taking was pivotal in identifying prolonged intermittent fasting as the underlying risk factor.

Neuroimaging plays a crucial supportive role in diagnosis, particularly MRI, which has higher sensitivity than CT. Typical symmetric hyperintensities on T2-weighted and FLAIR sequences involving the thalami, mammillary bodies, and periaqueductal region are highly suggestive of WE, though normal imaging does not exclude the diagnosis [4-8]. In nonalcoholic WE, atypical MRI patterns, including cortical involvement, have been reported more frequently than in alcoholic cases, possibly reflecting differences in pathophysiology or timing of presentation [2,3]. In this patient, the presence of classic MRI findings reinforced the clinical diagnosis and excluded structural or vascular etiologies.

Management of WE requires immediate parenteral thiamine replacement, as oral absorption may be unreliable in the acute setting. Although optimal dosing regimens remain debated, high-dose intravenous thiamine (≥500 mg three times daily initially) is widely recommended to ensure adequate central nervous system penetration [2-6]. Concomitant correction of electrolyte abnormalities, particularly magnesium deficiency, is essential, as magnesium is a cofactor for thiamine-dependent enzymes and its deficiency can blunt treatment response. Nutritional rehabilitation and long-term oral supplementation are critical to prevent recurrence.

The widespread promotion of fasting regimens on digital media and wellness platforms often lacks crucial warnings about nutritional risks, creating a significant public health concern [8,9]. This case illustrates a direct consequence of such unsupervised dietary practices. While evidence suggests potential metabolic benefits from controlled intermittent fasting, these benefits are contingent upon maintaining overall nutritional adequacy, particularly of micronutrients like thiamine. The dissociation of fasting practices from essential nutritional education places a subset of motivated individuals at inadvertent risk. Therefore, alongside clinical vigilance, there is a pressing need for collaborative efforts between healthcare providers, public health authorities, and media influencers to disseminate balanced, evidence-based guidelines [5,8].

## Conclusions

This case underscores the importance of maintaining a high index of suspicion for WE in patients presenting with subacute neurological symptoms, even in the absence of alcohol use or traditional risk factors. As demonstrated, prolonged intermittent fasting and extreme dietary practices can precipitate clinically significant thiamine deficiency, leading to potentially reversible but life-threatening neurological injury. Early recognition based on clinical features, supported by characteristic neuroimaging findings, and immediate initiation of high-dose parenteral thiamine are critical to favorable outcomes. The take-home message is that WE is a clinical diagnosis that should be considered in any malnourished or nutritionally vulnerable patient, regardless of etiology, and that prevention through nutritional education and appropriate supplementation is essential in the era of popularized weight loss regimens.

## References

[1] Namboodiri S, Rao S (2024). A case of prolonged Wernicke's encephalopathy after treatment with IV thiamine due to the subsequent development of refeeding syndrome. Cureus.

[2] Mizui R, Takada R, Ikuno K, Araki S, Okada T (2025). A case of prolonged Wernicke's encephalopathy successfully treated with long-term high-dose thiamine. Cureus.

[3] Anton SD, Moehl K, Donahoo WT (2018). Flipping the metabolic switch: understanding and applying the health benefits of fasting. Obesity (Silver Spring).

[4] Basouny N, Spigos J, Khvolis D (2023). Wernicke encephalopathy in a pediatric patient with avoidant restrictive food intake disorder: a rare presentation of thiamine deficiency. Am J Case Rep.

[5] Lian X, Wu M, Fan H (2020). Wernicke's encephalopathy due to malnutrition and parenteral nutrition in a patient with cerebral infarction: a case report. Medicine (Baltimore).

[6] Kohnke S, Meek CL (2021). Don't seek, don't find: The diagnostic challenge of Wernicke's encephalopathy. Ann Clin Biochem.

[7] Scalzo SJ, Bowden SC, Ambrose ML (2015). Wernicke-Korsakoff syndrome not related to alcohol use: a systematic review. J Neurol Neurosurg Psychiatry.

[8] Sechi G, Serra A (2007). Wernicke's encephalopathy: new clinical settings and recent advances in diagnosis and management. Lancet Neurol.

[9] Habas E, Farfar K, Errayes N (2023). Wernicke encephalopathy: an updated narrative review. Saudi J Med Med Sci.

